# Analysis of multi-omics differences in left-side and right-side colon cancer

**DOI:** 10.7717/peerj.11433

**Published:** 2021-05-12

**Authors:** Yanyi Huang, Jinzhong Duanmu, Yushu Liu, Mengyun Yan, Taiyuan Li, Qunguang Jiang

**Affiliations:** 1 Department of Gastrointestinal Surgery, First Affiliated Hospital of Nanchang University, Nanchang, Jiangxi, China; 2 Nanchang University, The Second Clinical Medicine College, Nanchang, Jiangxi, China; 3 Nanchang University, The First Clinical Medicine College, Nanchang, Jiangxi, China

**Keywords:** Left-side colon cancer, Right-side colon cancer, Mutation, Gene expression, Prognosis, Immune microenvironment

## Abstract

**Background:**

Colon cancer is one of the most common tumors in the digestive tract. Studies of left-side colon cancer (LCC) and right-side colon cancer (RCC) show that these two subtypes have different prognoses, outcomes, and clinical responses to chemotherapy. Therefore, a better understanding of the importance of the clinical classifications of the anatomic subtypes of colon cancer is needed.

**Methods:**

We collected colon cancer patients’ transcriptome data, clinical information, and somatic mutation data from the Cancer Genome Atlas (TCGA) database portal. The transcriptome data were taken from 390 colon cancer patients (172 LCC samples and 218 RCC samples); the somatic mutation data included 142 LCC samples and 187 RCC samples. We compared the expression and prognostic differences of LCC and RCC by conducting a multi-omics analysis of each using the clinical characteristics, immune microenvironment, transcriptomic differences, and mutation differences. The prognostic signatures was validated using the internal testing set, complete set, and external testing set (GSE39582). We also verified the independent prognostic value of the signature.

**Results:**

The results of our clinical characteristic analysis showed that RCC had a significantly worse prognosis than LCC. The analysis of the immune microenvironment showed that immune infiltration was more common in RCC than LCC. The results of differential gene analysis showed that there were 360 differentially expressed genes, with 142 upregulated genes in LCC and 218 upregulated genes in RCC. The mutation frequency of RCC was generally higher than that of LCC. BRAF and KRAS gene mutations were the dominant genes mutations in RCC, and they had a strong mutual exclusion with APC, while APC gene mutation was the dominant gene mutation in LCC. This suggests that the molecular mechanisms of RCC and LCC differed. The 4-mRNA and 6-mRNA in the prognostic signatures of LCC and RCC, respectively, were highly predictive and may be used as independent prognostic factors.

**Conclusion:**

The clinical classification of the anatomic subtypes of colon cancer is of great significance for early diagnosis and prognostic risk assessment. Our study provides directions for individualized treatment of left and right colon cancer.

## Introduction

Colon cancer is one of the most common cancers in the world and it is the second leading cause of cancer-related deaths in the United States (Siegel et al., 2020). The location of the tumor itself has not received much attention due to the belief that accurately locating the tumor would not affect patient survival.

However, in the past decade the differences between LCC and RCC have received more attention (Mik et al., 2017). The embryonic origin may help explain the genesis of this disease (Bufill, 1990). RCC is known to originate from the midgut, which includes the cecum, ascending colon, and hepatic flexure. In contrast, LCC originates from the hindgut, which includes the splenic flexure, descending colon, and sigmoid colon.

LCC and RCC have received increased attention because of clear differences in their prognosis, outcomes, and clinical response to chemotherapy. It has been reported that LCC is associated with a better prognosis compared with RCC (Kalantzis et al., 2020). A recent systematic review noted that many studies have identified differences in their epidemiology, clinical presentation, pathology, and genetic mutations through anatomical subsites (Imperial et al., 2018).

Most of the studies indicated that patients with RCC showed lower survival rates compared with LCC (Nakagawa-Senda et al., 2019). However, the data are still controversial. Weiss et al. demonstrated that when analysis was adjusted for multiple variables, including patient, disease, comorbidity, and treatment, there was no overall difference in the 5-year mortality between LCC and RCC (Weiss et al., 2011).

Additional studies on LCC and RCC are needed. We performed a multi-omics analysis of LCC and RCC using clinical characteristics, the immune microenvironment, transcriptomic differences, and mutation differences to determine the importance of classifying these anatomic colon cancer subtypes.

## Method and data

### Data collection and preprocessing

First, we downloaded colon cancer patients’ transcriptome data, clinical information, and somatic mutation data from the Cancer Genome Atlas (TCGA) database (https://portal.gdc.cancer.gov). The transcriptome data were comprised of 390 colon cancer patients (172 LCC samples and 218 RCC samples), and the somatic mutation data were comprised of 329 colon cancer patients (142 LCC samples and 187 RCC samples). According to Dwertmann et al., the LCC consists of the descending colon, sigmoid colon, and splenic flexure of colon and the RCC consists of the ascending colon, cecum, and hepatic flexure of colon (Hsu et al., 2019). We used genecode.v22.annotation (https://www.gencodegenes.org/) to comment on the transcriptional data downloaded from TCGA database.

### Clinical analysis in LCC and RCC

We used R to classify the data used to analyze the differences between LCC and RCC in terms of age, gender, pT, pN, pM, pStage and survival. Pearson’s chi-square (χ2) test was used to calculate differences in clinical characteristics between LCC and RCC. We compared the overall survival rates of LCC and RCC in different clinical subtypes using the survival package in R.

### Immune microenvironment in LCC and RCC

We obtained immune-related gene sets with 29 immune cell types and immune-related functions from previous studies (Bedognetti et al., 2015; Ren et al., 2020; Liu et al., 2018; Maibach et al., 2020; Aran et al., 2016) to explore the differences between LCC and RCC in the immune microenvironment. We used the single sample gene set enrichment analysis (ssGSEA) algorithm to obtain the scores of 29 immune cell types and immune-related functions with the ‘GSVA’ package in R (Hänzelmann, Castelo & Guinney, 2013). We visualized the results using the pheatmap package in R (Galili et al., 2018). We used the estimate package in R to analyze the differences between LCC and RCC in the immune microenvironment to calculate the immune score, stromal score, ESTIMATE score, and tumor purity. Then we compared the differences between the two groups using the Mann–Whitney *U* test. We also compared the expression levels of the human leukocyte antigen (HLA) gene family and immune check-point genes, and the abundance of immune cell infiltration in LCC and RCC. We obtained the immune cell infiltration data using CIBERSORT (Chen et al., 2018).

### Screening differential genes in LCC and RCC

We used the |log2 fold change(LogFC)|>1 and the false discovery rate (FDR) <0.05 with the Wilcox test to identify the differences in expression of mRNAs in LCC and RCC. The results were visualized using heatmap and volcano diagrams. Gene Ontology (GO) and the Kyoto Encyclopedia of Genes and Genomes (KEGG) were used to determine the enrichment of the differential genes through the Database for Annotation, Visualization and Integrated Discovery (DAVID) (https://david.ncifcrf.gov/) (Yang et al., 2019). The top 20 biological processes (BP) of GO enrichment analysis (Chen et al., 2017) were depicted in a circle diagram, and the top 15 KEGG pathways (Altermann & Klaenhammer, 2005) were depicted in a bubble diagram.

### Screening prognostic mRNAs in LCC and RCC by univariate COX analysis

We included different genes in our study. We used the survival package in R with *P* < 0.005 to identify the prognostic mRNAs in LCC and RCC, respectively, using univariate COX (Tibshirani, 2009). According to univariate COX analysis, there were 22 genes associated with the prognosis of RCC, with a potential collinear relationship among them. We used the LASSO regression algorithm with a penalty term to delete genes with multicollinearity for additional analysis.

### Construction and verification of the prognostic signature and validation of prognostic models

Prognostic genes related to LCC and RCC were included in our study. We set up a random number seed in order to divided LCC patients from TCGA into a training set and an internal testing set with a 1:1 ratio and established a 4-mRNA LCC prognosis model using multivariate COX regression analysis with a noose penalty (Grant, Hickey & Head, 2019; Zhou et al., 2019). We used the same method to establish a 6-mRNA RCC prognosis signature (Marisa et al., 2013; Zhang et al., 2016; Zhang et al., 2020). The samples was divided into two groups using the median risk score. We judged the efficacy of the model by plotting the Kaplan–Meier (KM) curve and receiver operating characteristic (ROC) curve (Obuchowski & Bullen, 2018). The GSE39582 data set was downloaded from the Gene Expression Omnibus (GEO) database (https://www.ncbi.nlm.nih.gov/geo/query/acc.cgi?acc=GSE39582) (Barrett et al., 2013) and was used as an external validation set. We validated the model by plotting the Kaplan–Meier (KM) curve. The GSE39582 data set included 566 colon cancer samples (342 LCC samples and 224 RCC samples) and their corresponding survival information in accordance with the GPL570 (Affymetrix Human Genome U133 Plus 2.0 Array) (Table S1). We performed an independent prognostic analysis of the risk score in the total TCGA set to further verify the model’s efficacy. The risk score was calculated as (Cho et al., 2019):

(1) $$Riskcore=\sum_{i=1}^{N}(Expi\times Coef)$$

with N representing the number of signature genes, Expi representing the gene expression levels, and Coef representing the estimated regression coefficient value from the Cox proportional hazards analysis.

### Single gene mutation analysis in LCC and RCC

On the JAVA8 platform, we analyzed the number of variants and the length of exons for each sample using Perl scripts to calculate mutation frequency (Tabibzadeh et al., 2020). Samples were divided into two groups according to the location of colon cancer and the Mann–Whitney test (McGee, 2018) was used to compare the tumor mutation burden (TMB) difference between two groups. We used the maftools package (Mayakonda et al., 2018) for visualization and performed Fisher’s exact test in pairs between the top 25 mutated genes to analyze the mutational exclusion and co-occurrence. We also used oncoplot in R to visualize the top 30 mutated genes from the 142 LCC samples and 187 RCC samples to produce waterfall plots. Then we used the ggplot2 and boxplot packages to visualize the classification and frequency of mutation types, frequency of variant types, frequency of SNV classes, the tumor mutation burden in specific samples, and the top 10 mutated genes in LCC and RCC. The top 10 mutated genes in LCC were: *APC*, *TP53*, *TTN*, *KRAS*, *MUC16*, *SYNE1*, *FAT4*, *RYR2*, *PIK3CA*, and *OBSCN*. The top 10 mutation genes in RCC were: *TTN*, *APC*, *MUC16*, *SYNE1*, *TP53*, *KRAS*, *FAT4*, *PIK3CA*, *PCLO*, and *ZFHX4*.

## Results

### Differences in clinical characteristics between LCC and RCC

**Figure 1:**
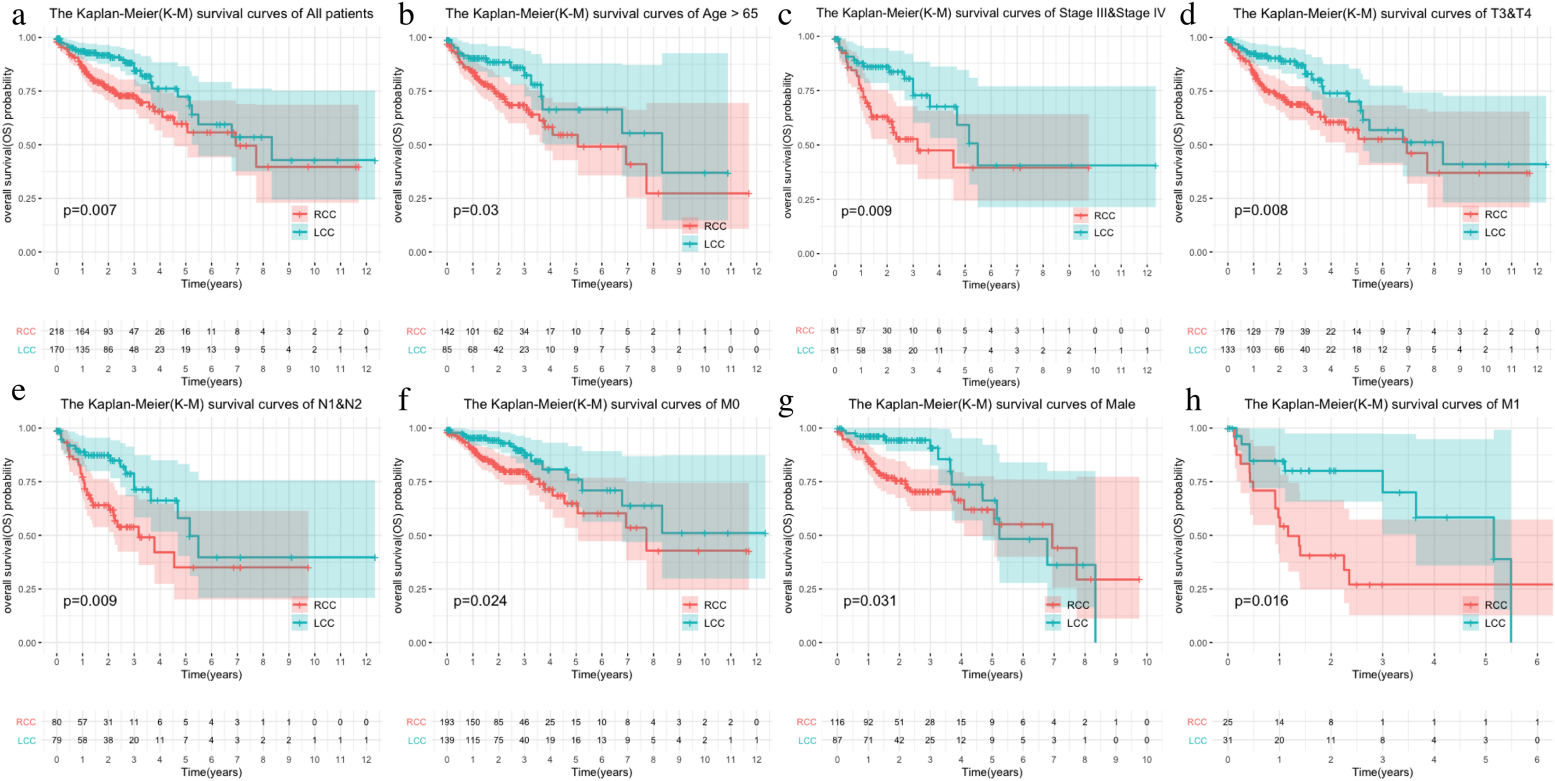
Comparison of survival rates of LCC and RCC in different clinical subtypes. Survival analysis of different clinical characteristics including (A) all patients, (B) Age > 65, (C) Stage III & Stage IV, (D) T3&T4, (E) N1&N2, (F) M0, (G) Male, (H) M1.

The LCC and RCC data in the TCGA database and the results of the chi-square test on clinical characteristics are shown in Table 1. We classified the data by stage, T, N, M, and age after separating the data by LCC and RCC. We used the Kaplan–Meier (KM) curve of over survival (OS) to compare the survival differences of different clinical characteristics between the two groups. The results indicated that RCC had a worse prognosis than LCC, which was also seen in stages III-IV, T3-4, and N1-2 (Figs. 1A–1D). The survival rate of RCC was worse than that of LCC (Figs. 1E–1F) although there was no statistical difference between the M1 and age > 65 subgroups.

**Table 1:** Clinical features for the COAD patients in the LCC and RCC in TCGA.

Parameters	LCC patients (*n* = 172)	RCC patients (*n* = 218)	χ^2^	*P* value
**Age, y**			7.814	0.005
≤65	85	76		
>65	87	142		
**Gender**			0.035	0.852
Male	89	116		
Female	83	102		
**pT**			0.263	0.608
T1-2	37	41		
T3-4	135	176		
unknow	0	1		
**pN**			3.022	0.082
N0	93	138		
N1-2	79	80		
**pM**			2.099	0.147
M0	126	162		
M1	31	25		
unknow	15	31		
**pStage**			2.934	0.087
Stage I–II	89	130		
Stage III–IV	81	81		
unknow	2	7		
**Survival**			5.122	0.024
Alive	144	163		
Dead	26	55		
unknow	2	0		

**Note:**

LCC: Left-side colon cancer; RCC: Right-side colon cancer; TCGA: The Cancer Genome Atlas; χ^2^: Chi-square value.

### Immune microenvironment landscape between LCC and RCC

**Figure 2:**
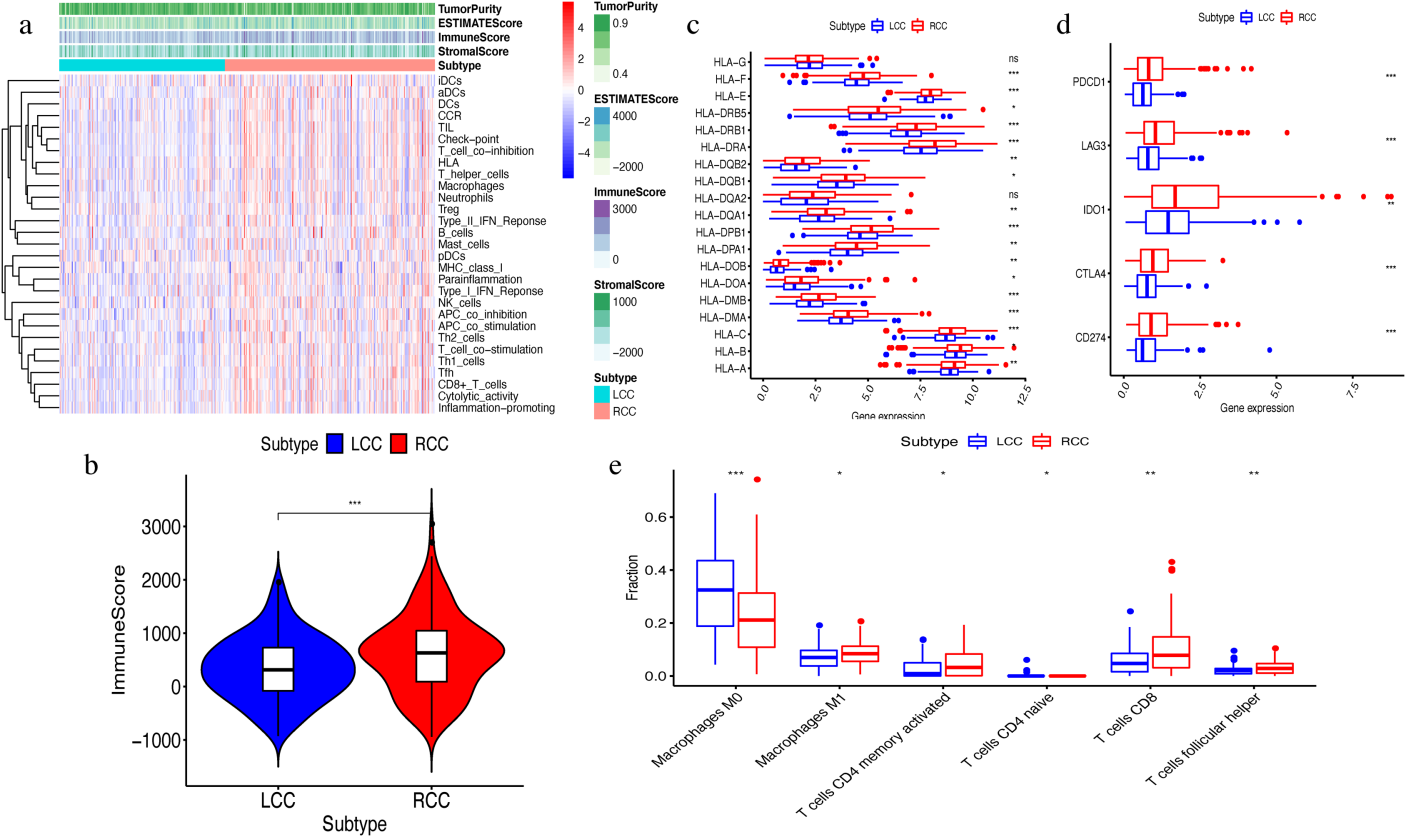
Exploration and validation the differences of immune microenvironment between LCC and RCC. Through ssGSEA, 29 immune-related gene sets were enriched, including immune cells and immune processes. (A) The heat map is also included the tumor purity, ESTIMATE score, immune score and stromal score. (B) Variance analysis of the immune score between LCC and RCC. (C) The expression levels of HLA gene family in samples from LCC and RCC. (D) The expression levels of immune checkpoint genes (PDCD1, LAG3, IDO-1, CTLA4, CD274) in samples from LCC and RCC. (E) The abundance of six types of infiltrating immune cells in samples from LCC and RCC. *P < 0.05, **P < 0.01, ***P < 0.001.

The ssGSEA algorithm showed that 29 types of immune cells and their functions were enriched in each sample. We then obtained the immune score, stromal score, ESTIMATE score, and tumor purity. The heatmap indicated that the RCC had a higher immune invasion than the LCC (Fig. 2A). Comparing the two groups’ scores, we found that only the immune scores were significantly different (Fig. 2B). We confirmed that RCC had a higher immune infiltration than LCC by further comparing the expression levels of the *HLA* gene family and immune checkpoint-related genes and the abundance of immune cell infiltration (Figs. 2C–2D). Previous studies have shown that the changes in *HLA* class I genes in colon cancer are closely related to RCC, suggesting microsatellite instability (MSI). In addition, the high expression of *PD-L1* also occurs more frequently in RCC, indicating MSI (Kikuchi et al., 2019). Our results support the conclusion that RCC has more immune infiltration and is highly correlated with MSI. Therefore, this result suggests that right-side colon cancer was significantly more reactive than left-side colon cancer in immune response, which might provide new treatments for colon cancer.

### Differential gene analysis between LCC and RCC

**Figure 3:**
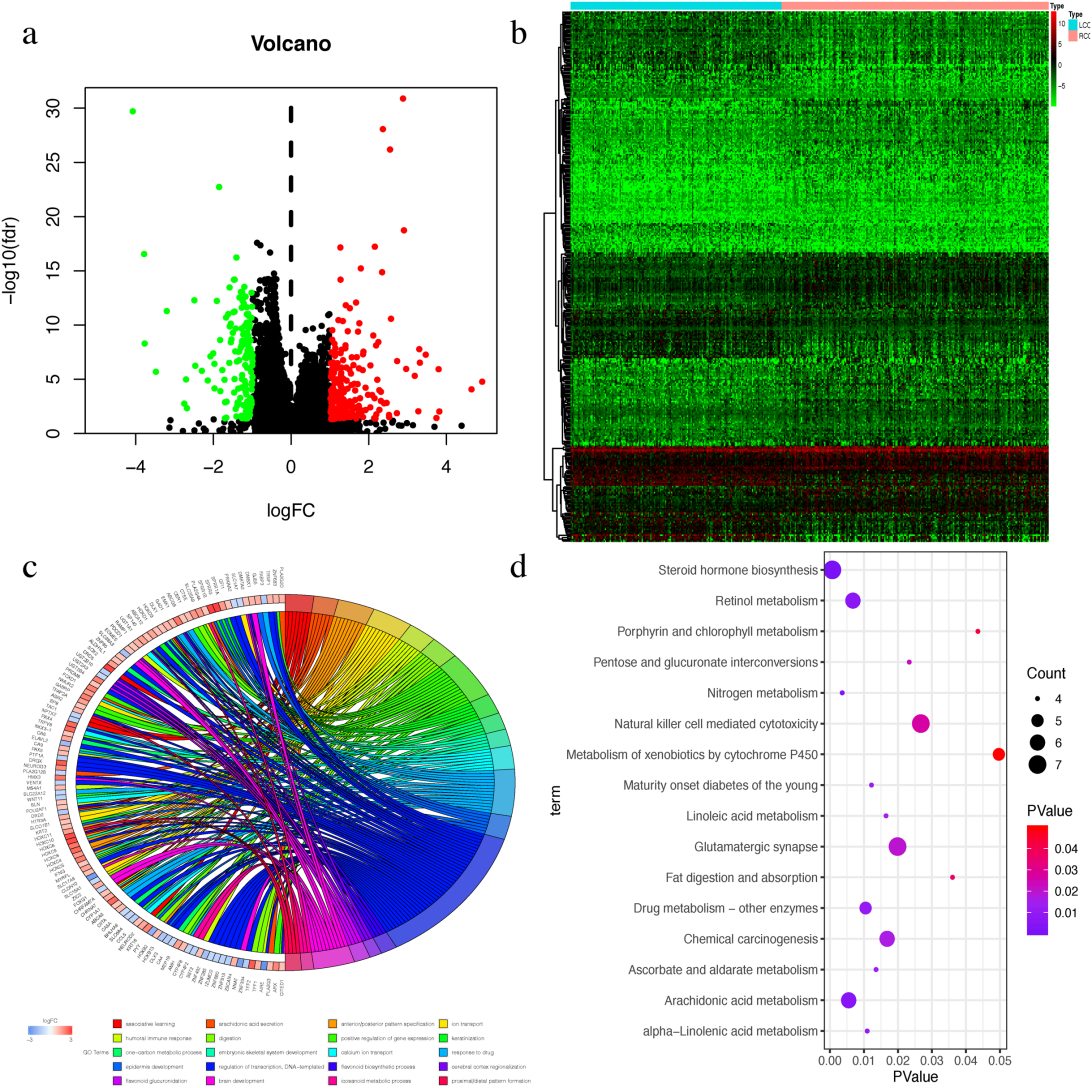
The Differential expressed mRNAs in LCC and RCC. (A) Volcano plot for differential expressed mRNAs in LCC and RCC. Green dots represent up-regulated genes in LCC, while red dots represent up-regulated genes in RCC. (B) Heatmap of differential expressed mRNAs between LCC and RCC. (C) Circle diagram demonstrated the top 20 biological processes of GO enrichment analysis. (D) Bubble diagram demonstrated the top 15 KEGG pathways.

The Wilcox test was used to extract differential mRNAs to obtain 360 differential genes, which included 218 up-regulated genes in RCC and 142 up-regulated genes in LCC (Figs. 3A–3B). All of the differential genes are shown in Table S2. All of the differentially expressed genes were enriched by the biological processes of GO and KEGG pathways in the DAVID database (Tables S3 and S4). The top 20 biological processes of GO enrichment analysis were graphed in a circle diagram, while the top 15 KEGG pathways were displayed in a bubble diagram (Figs. 3C–3D). The top three biological pathways were ‘associative learning’, ‘arachidonic acid secretion’, and ‘anterior/posterior pattern specification’. The differentially expressed genes were significantly enriched in the steroid hormone biosynthesis pathway.

### Univariate COX screening of prognostic genes in LCC and RCC

We screened the genes related to the prognosis of LCC and RCC using univariate Cox analysis in the LCC and RCC patients with *P* < 0.005. We obtained 9 genes related to prognosis in LCC and 22 genes related to prognosis in RCC (Tables 2–3). In order to avoid model overfitting, we performed LASSO regression analysis with the penalty term on RCC to solve the multicollinearity problem again by dimension reduction, and finally obtained 12 genes related to prognosis in RCC (Fig. 5B).

**Table 2:** The prognostic mRNAs by univariable Cox analysis in LCC.

mRNA	HR	95% CI		*P* value
		Low	High	
C1orf105	1.412	1.154	1.729	0.001
OSR1	10.074	2.908	34.898	<0.001
FAM132B	3.310	1.597	6.858	0.001
WNT7A	1.750	1.212	2.525	0.003
FDCSP	1.083	1.026	1.144	0.004
SMTNL2	1.317	1.109	1.564	0.002
FCER2	1.446	1.144	1.828	0.002
TNNT1	1.114	1.050	1.182	<0.001
RSPO4	1.230	1.074	1.410	0.003

**Note:**

LCC: Light-side colon cancer; mRNA: messenger RNA; CI: confidence interval; HR: hazard ratio.

**Table 3:** The prognostic mRNAs by univariable Cox analysis in RCC.

mRNA	HR	95% CI		*P* value
		Low	High	
LMX1A	3.964	1.712	9.176	0.001
COLGALT2	1.165	1.070	1.270	0.000
SNCB	2.748	1.537	4.912	0.001
OFCC1	48.830	6.787	351.331	0.000
FABP7	4.518	1.896	10.770	0.001
PAX4	1.130	1.045	1.223	0.002
KLRG2	1.253	1.124	1.398	<0.001
PAX5	1.347	1.126	1.612	0.001
PCDH8	166.438	7.157	3,870.700	0.001
HS6ST3	4.836	1.677	13.953	0.004
SYNGR3	1.116	1.045	1.191	0.001
CHST6	1.266	1.111	1.443	<0.001
SLC22A31	1.635	1.324	2.020	<0.001
NEUROD2	1.877	1.299	2.712	0.001
TCAP	1.791	1.250	2.566	0.001
GREB1L	2.482	1.369	4.502	0.003
FCER2	1.118	1.042	1.198	0.002
SLC7A10	2.237	1.370	3.653	0.001
APLP1	1.087	1.029	1.147	0.003
RSPO4	1.479	1.216	1.799	<0.001
INSM1	1.038	1.012	1.064	0.004
CCDC160	1.436	1.200	1.718	<0.001

**Note:**

RCC: Right-side colon cancer; mRNA: messenger RNA; CI: confidence interval; HR: hazard ratio.

### Construction of prognosis signature in LCC and RCC

TCGA LCC patients were divided into a training set and an internal testing set at a 1:1 ratio. Multivariate COX regression analysis with noose penalty was then used to establish a 4-mRNA LCC prognosis signature and a 6-mRNA RCC prognosis signature.

**Figure 4:**
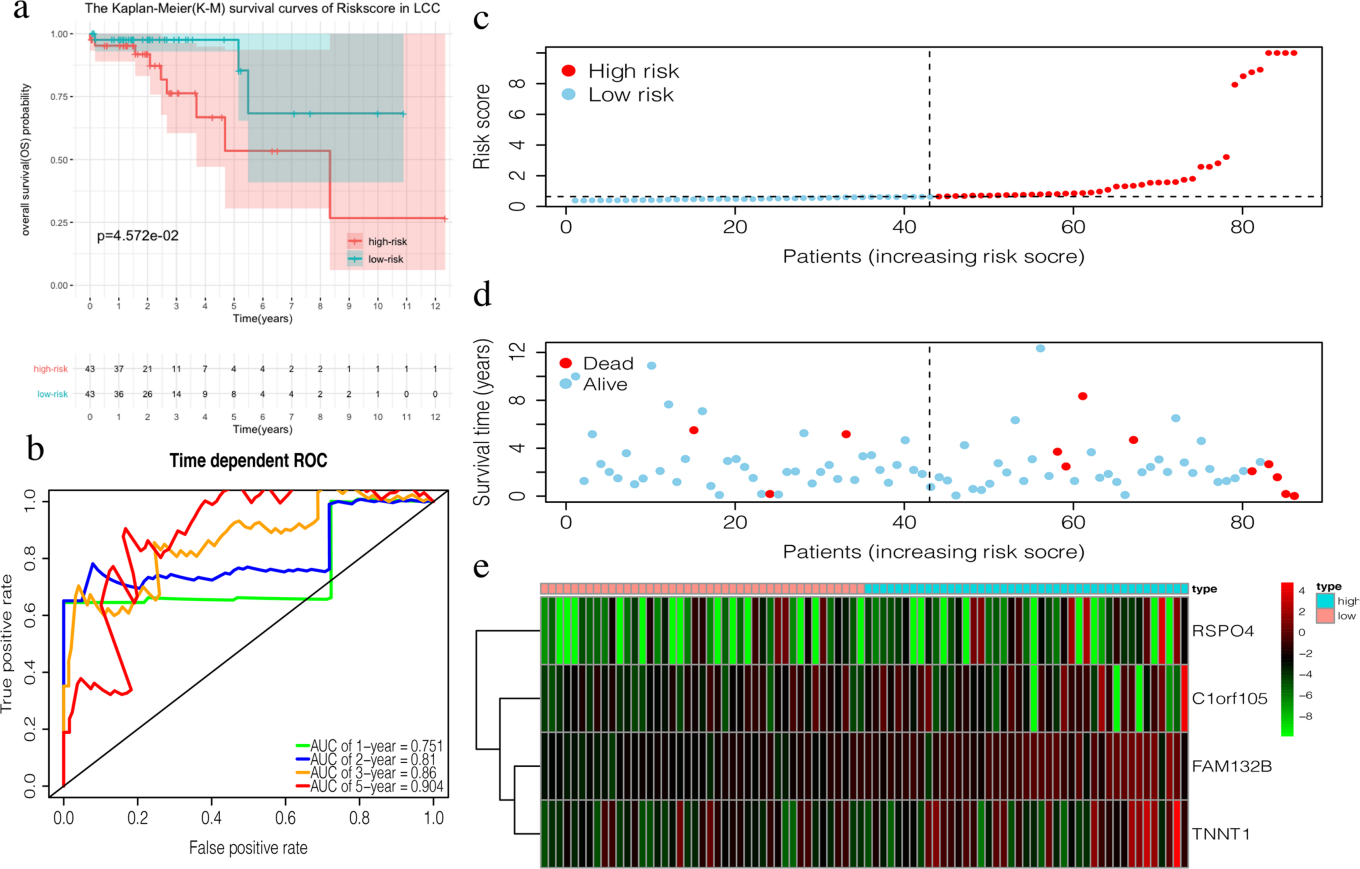
Construction of the prognostic model in the training group of LCC. (A) The Kaplan–Meier (K–M) survival curves in the training set, (B) Time-dependent ROC curves in the training set at 1-year, 2-year, 3-year and 5-year. (C) The survival status of LCC patients in the training group. Green dots represent the patient is still alive, while red dots represent the patient has dead. (D) Risk scores of LCC patients in the training group. Green dots represent the patient assigned to the low risk group, while red dots represent the patient assigned to the high risk group. (E) mRNAs expression levels of four mRNA LCC prognosis signature in the training group.

The 4-mRNA LCC prognosis signature and risk score were calculated as: C1orf105*0.458+FAM132B*1.703+TNNT1*0.130+RSPO4*0.268 (Table 4). The median risk score (0.622) in the training set was used to assign patients to the high risk or low risk group. Patients with a high risk score had significantly worse survival rates than those with low-risk scores (*P* = 0.046, Fig. 4A). Furthermore, the AUC of the risk score for 1-year, 2-year, 3-year, and 5-year OS were 0.751, 0.810, 0.860, and 0.904, respectively (Fig. 4B). The survival status, risk scores, and gene expression data of LCC patients in the training group are shown in Figs. 4C–4E. *RSPO4*, *FAM132B*, and *TNNT1* were highly expressed in the high-risk group, while *C1orf105* was not well-expressed in the high-risk group.

**Table 4:** Multivariate Cox regression modeling in LCC.

id	coef	HR	95% CI		*P* value
			Low	High	
C1orf105	0.458	1.581	1.134	2.205	0.007
FAM132B	1.703	5.492	1.390	21.693	0.015
TNNT1	0.130	1.139	1.026	1.265	0.015
RSPO4	0.268	1.307	1.087	1.572	0.004

**Note:**

LCC: Light-side colon cancer; CI: confidence interval; HR: hazard ratio.

**Figure 5:**
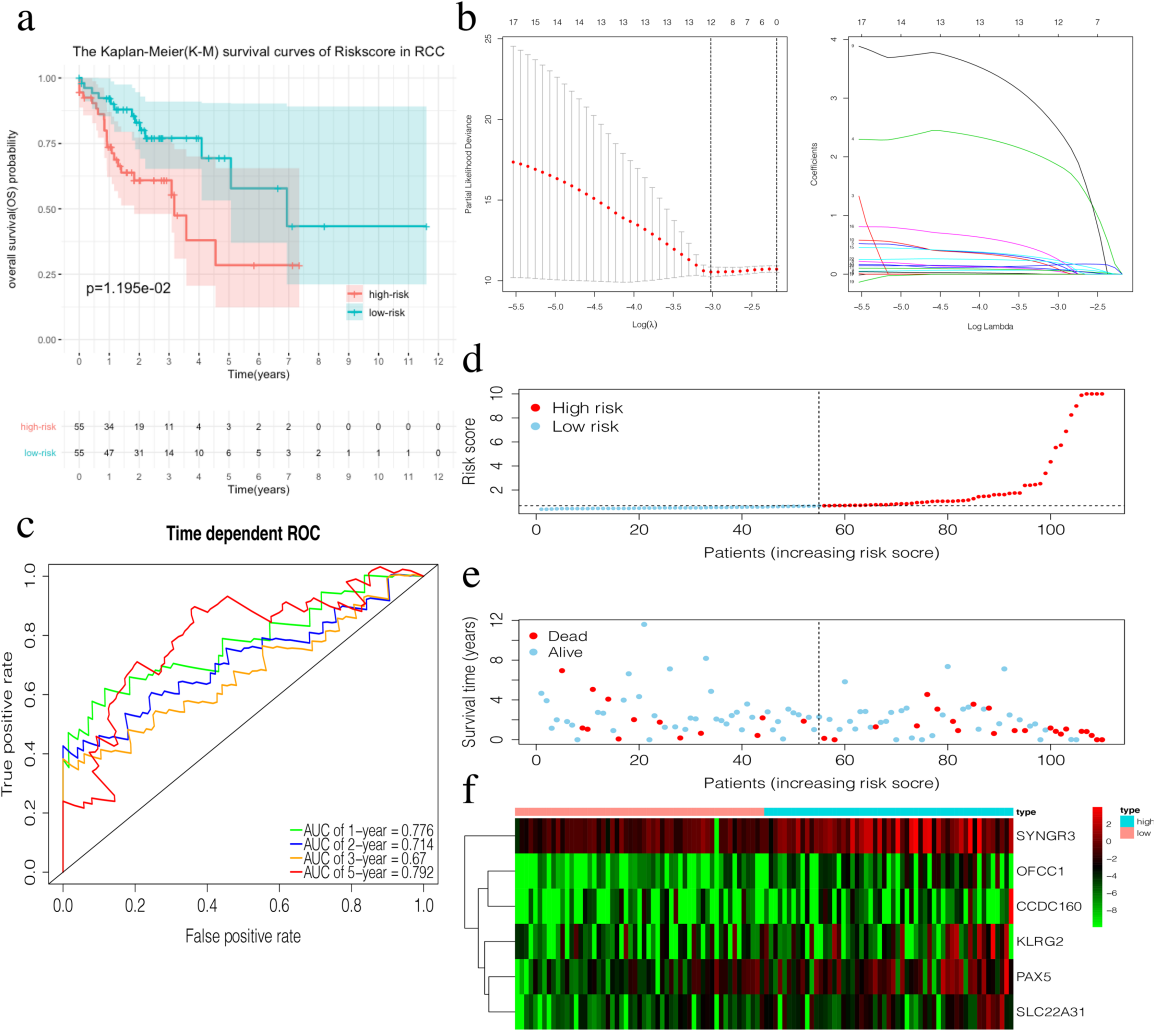
Construction of the prognostic model in the training group of RCC. (A) The Kaplan–Meier (K–M) survival curves in the training set. (B) Optimal parameters for Lasso Regression Analysis. (C) Time-dependent ROC curves in the training set at 1-year, 2-year, 3-year and 5-year. (D) The survival status of RCC patients in the training group. Green dots represent the patient is still alive, while red dots represent the patient has dead. (E) Risk scores of RCC patients in the training group. Green dots represent the patient assigned to the low risk group, while red dots represent the patient assigned to the high risk group. (F) mRNAs expression levels of six mRNA LCC prognosis signature in the training group.

The risk score of the 6-mRNA RCC prognosis signature was calculated as: OFCC1*4.834+KLRG2*0.195+PAX5*0.461+SYNGR3*0.096+SLC22A31*1.232+CCDC160*0.368 (Table 5). The median risk score (0.689) in the training set was used to assign patients to the high risk or low risk group. Patients with a high-risk score was had a significantly worse survival rate than those with a low-risk score (0.012, Fig. 5A). Furthermore, the AUC of the risk score for 1-year, 2-year, 3-year, and 5-year OS were 0.776, 0.714, 0.670, and 0.792, respectively (Fig. 5C). The survival status, risk scores, and gene expression data of RCC patients in the training group are shown in Figs. 5D–5F. All six genes were highly-expressed in the high-risk group.

**Table 5:** Multivariate Cox regression modeling in RCC.

id	coef	HR	95% CI		*P* value
			Low	High	
OFCC1	4.834	125.723	8.492	1861.210	<0.001
KLRG2	0.195	1.215	1.011	1.461	0.038
PAX5	0.461	1.586	1.201	2.095	0.001
SYNGR3	0.096	1.101	1.007	1.204	0.035
SLC22A31	1.232	3.428	1.599	7.350	0.002
CCDC160	0.368	1.444	1.213	1.720	<0.001

**Note:**

RCC: Right-side colon cancer; CI: confidence interval; HR: hazard ratio.

### Validation of the prognosis signature in LCC and RCC

The prognostic accuracy of the prognosis signature was validated in three independent cohorts, including the testing set, the total TCGA data set, and the GSE39582 data set.

**Figure 6:**
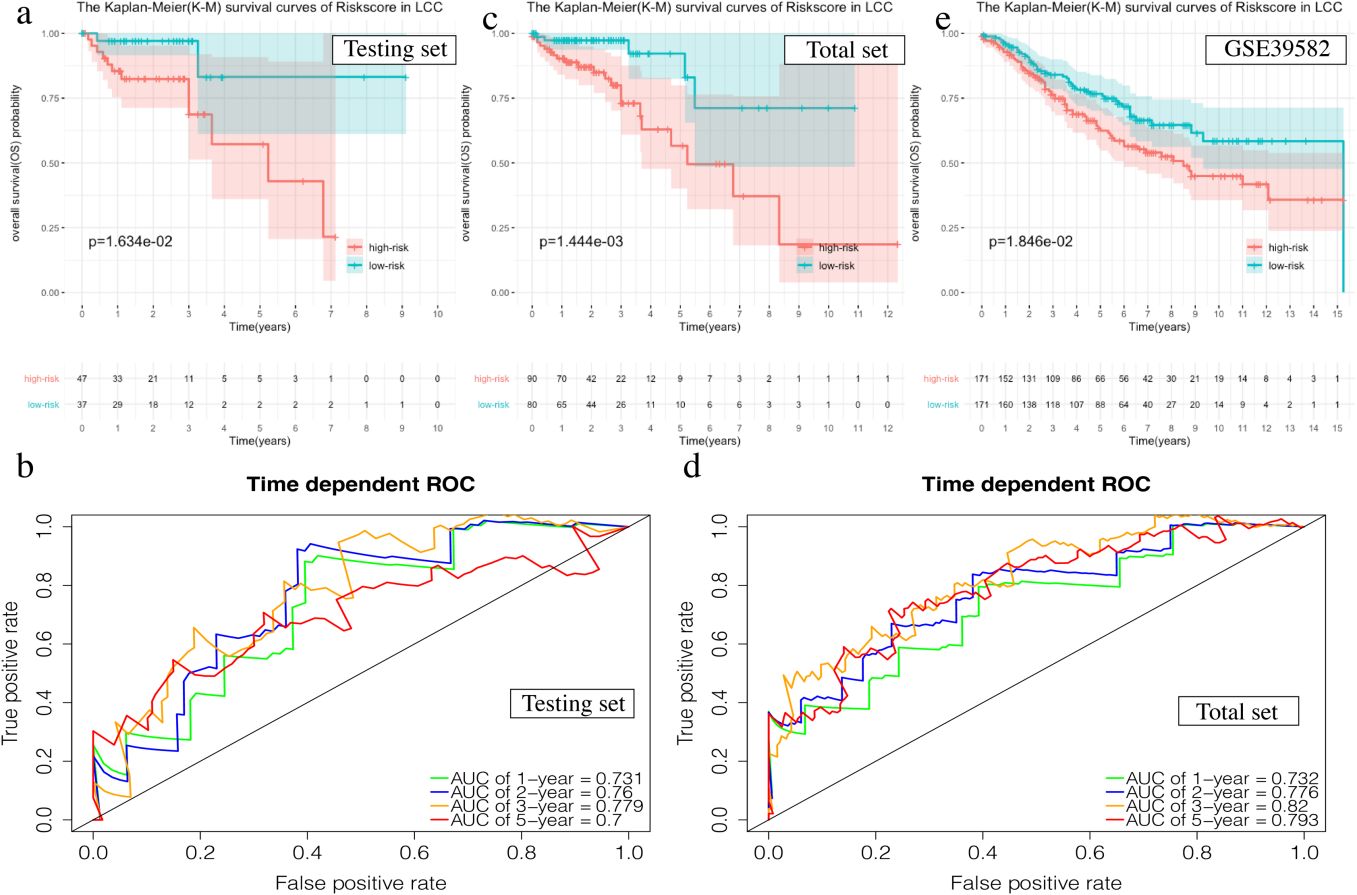
Validation of the prognostic signature of LCC. (A, C, E) The Kaplan–Meier (K–M) survival curves in the testing set, the total set and GSE39582. (B, D) Time-dependent ROC curves in the testing set and the total set at 1-year, 2-year, 3-year and 5-year.

The OS in the high-risk group was significantly worse than that of the low-risk group in the testing set in the 4-mRNA LCC prognosis signature (*P* = 0.016, Fig. 6A). The predicted 1-year, 2-year, 3-year, and 5-year OS was 0.731, 0.760, 0.779, and 0.700, respectively (Fig. 6B). The total TCGA set also validated the prognostic accuracy of the signature (*P* = 0.001, Fig. 6C), with respective AUCs of 0.732, 0.776, 0.820, and 0.793 for 1-year, 2-year, 3-year, and 5-year OS (Fig. 6D).

**Figure 7:**
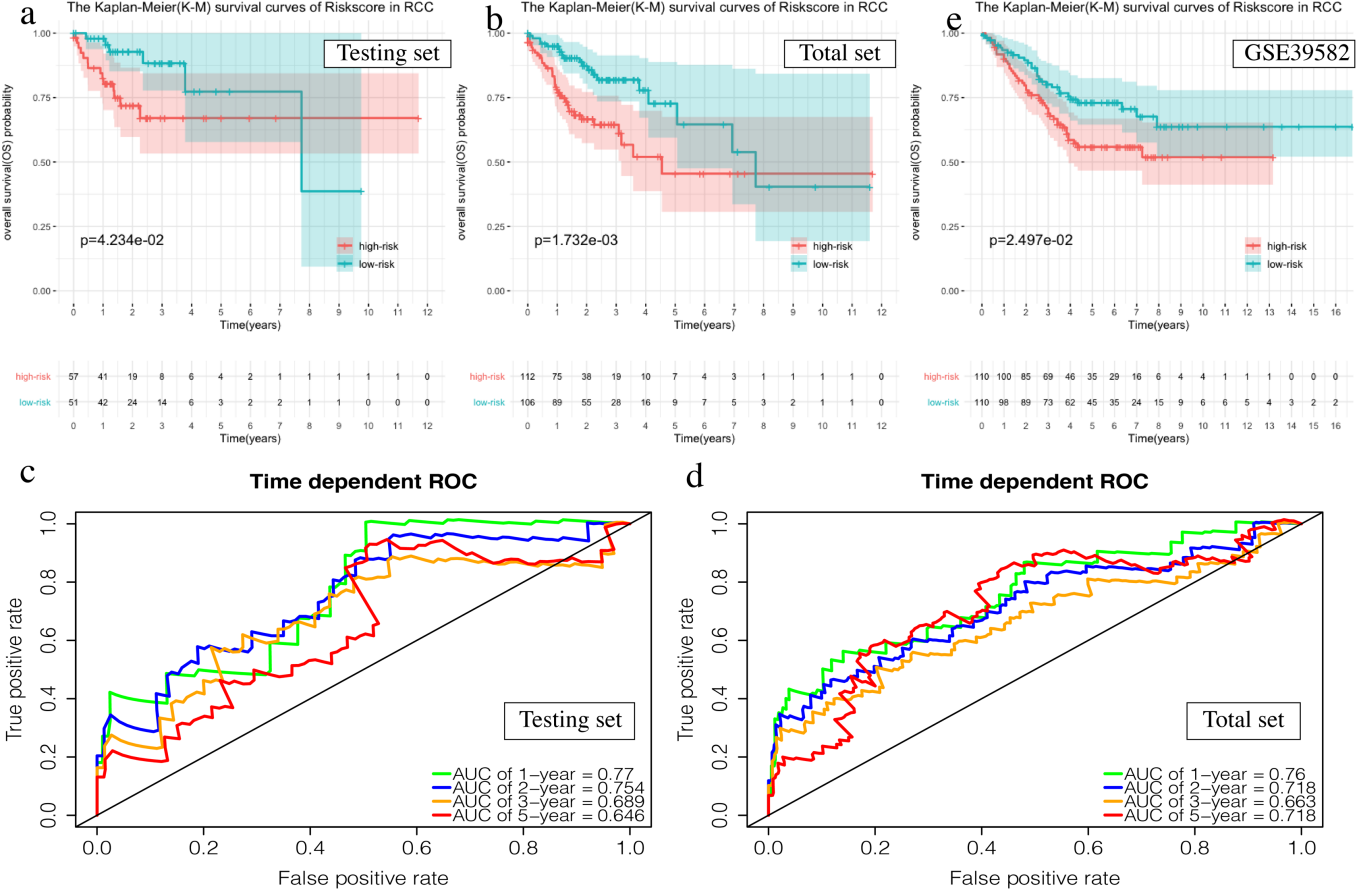
Validation of the prognostic signature of RCC. (A, C, E) The Kaplan–Meier (K-M) survival curves in the testing set, the total set and GSE39582. (B, D) Time-dependent ROC curves in the testing set and the total set at 1-year, 2-year, 3-year and 5-year.

The OS of the high-risk group was significantly worse than that of the low-risk group in the testing set for the 6-mRNA RCC prognosis signature (*P* = 0.042, Fig. 7A). The predicted 1-year, 2-year, 3-year, and 5-year OS was 0.770, 0.754, 0.689, and 0.646, respectively (Fig. 7B). The total TCGA set validated the prognostic accuracy of the signature (*P* = 0.002, Fig. 7C), with respective AUCs of 0.760, 0.718, 0.663, and 0.718 for 1-year, 2-year, 3-year, and 5-year OS (Fig. 7D).

The GSE39582 data set showed the same conclusion in the 4-mRNA LCC prognosis signature (*P* = 0.185) and the 6-mRNA RCC prognosis signature (*P* = 0.25) (*P* = 0.018, Fig. 6E; *P* = 0.025, Fig. 7E). The survival status, risk scores and gene expression data of LCC and RCC patients in the testing set and total TCGA set are shown in Figs. S2 and S3.

### The prognosis signature confers additional prognostic power for LCC and RCC patients

**Figure 8:**
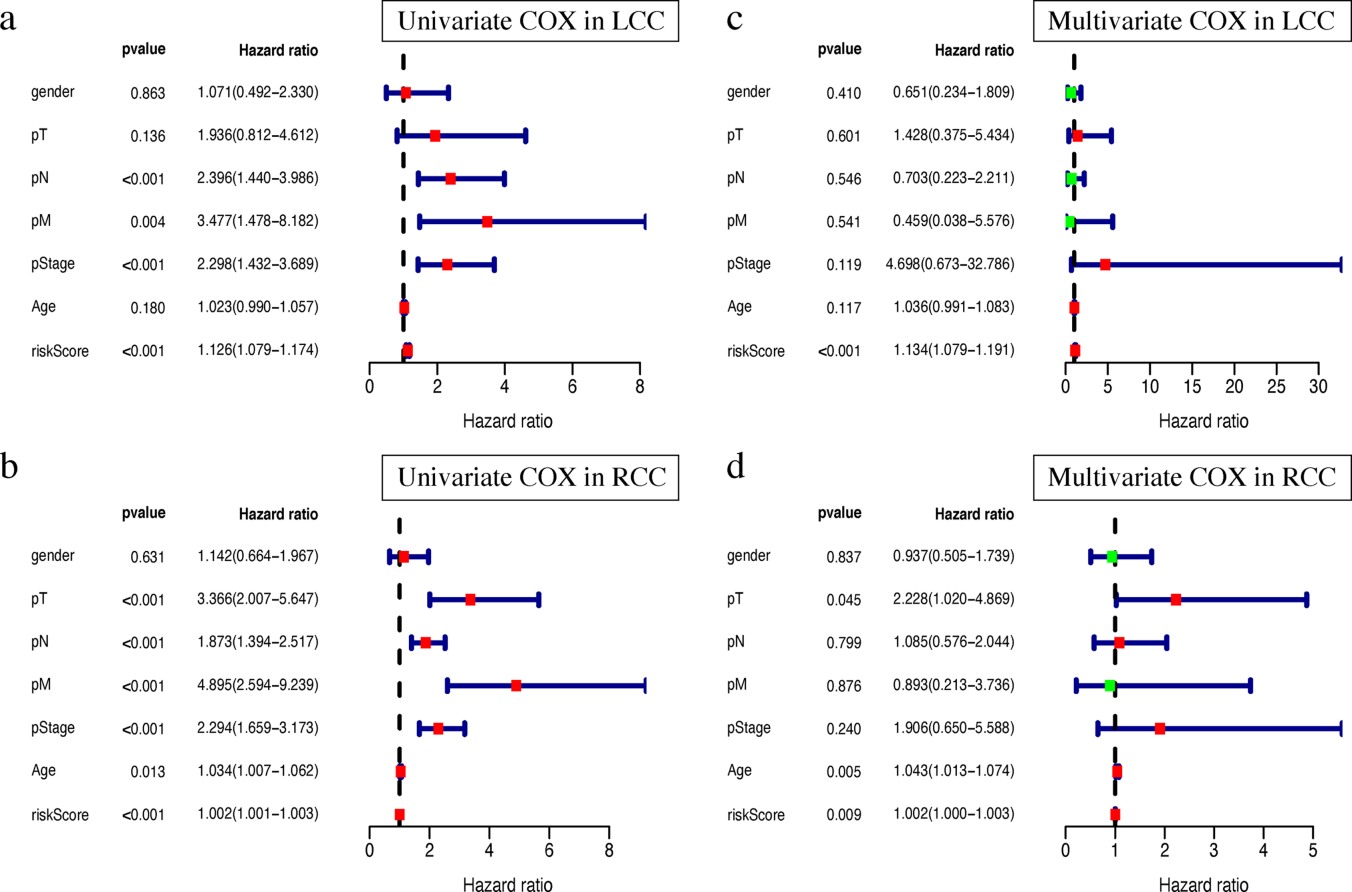
Independent prognostic analysis of two prognostic signatures. (A) Univariate COX analysis of LCC prognostic signatures and clinical characteristics. (B) Univariate COX analysis of RCC prognostic signatures and clinical characteristics. (C) Multivariate COX analysis of LCC prognostic signatures and clinical characteristics. (D) Multivariate COX analysis of RCC prognostic signatures and clinical characteristics.

Clinical characteristics, including the pStage (*P* < 0.001), pN (*P* < 0.001), pM (*P* = 0.004), and the risk score (*P* < 0.001), were closely associated with patient survival in LCC (Fig. 8A). The pStage (*P* < 0.001), pT (*P* < 0.001), pN (*P* < 0.001), pM (*P* < 0.001), age (*P* = 0.013), and risk score were closely associated with patient survival in RCC (Fig. 8B). Multivariate Cox regression analysis further showed that the our signature is an independent prognostic indicator for OS in LCC and RCC (Figs. 8C–8D, Tables S5–S6).

### Single gene mutation landscape in LCC and RCC

**Figure 9:**
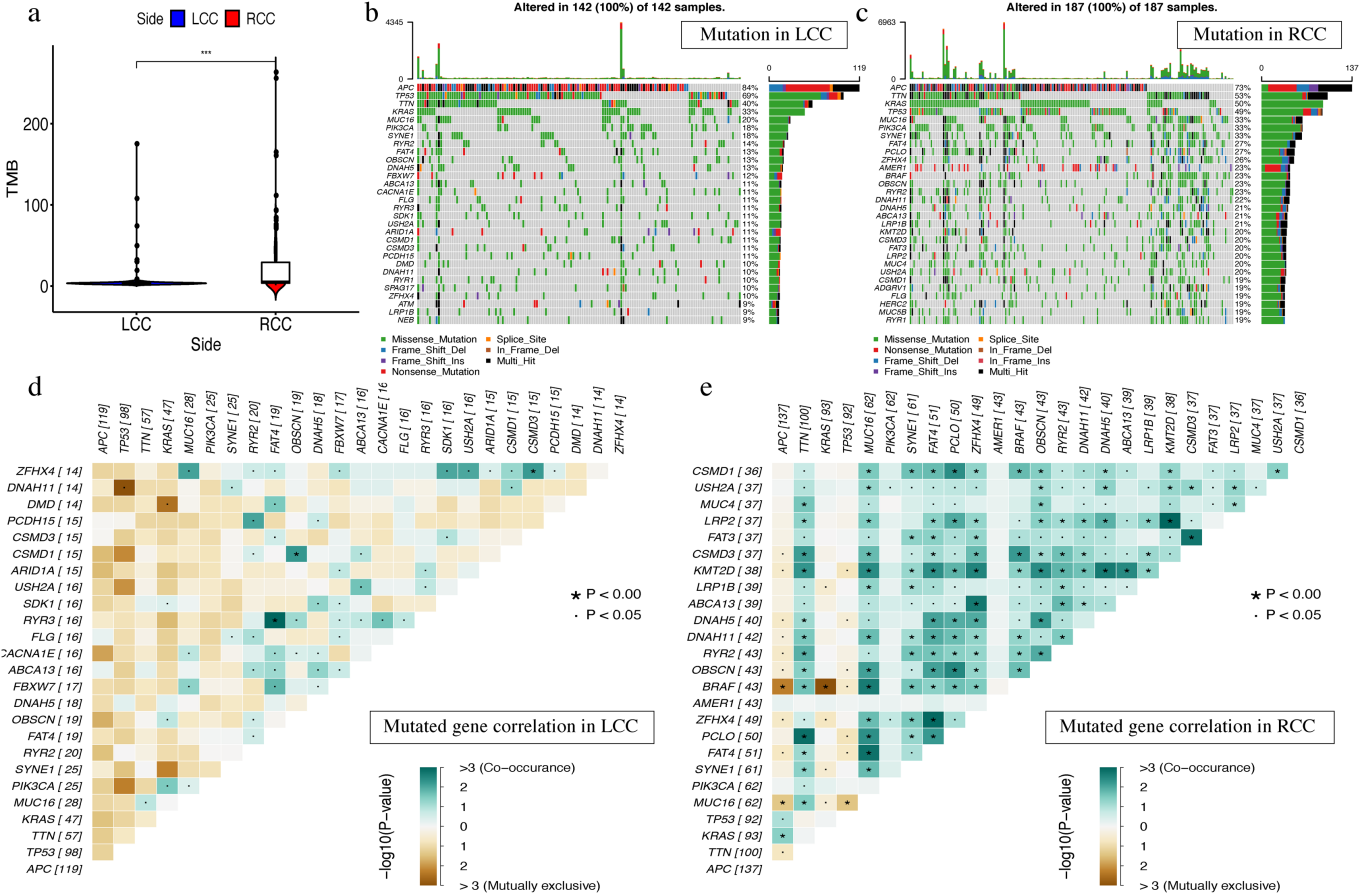
The landscape of single gene mutation in LCC and RCC. (A) The TMB of samples from two immune subgroups (*P < 0.05, **P < 0.01, ***P < 0.001) (B) Waterfall plot displayed the top 30 frequently mutated genes in LCC. (C) Waterfall plot displayed the top 30 frequently mutated genes in RCC. (D) The coincident and exclusive associations across mutated genes in LCC. (E) The coincident and exclusive associations across mutated genes in RCC.

The most obvious mutations, including missense mutations, were: deletion, nonsense mutation, splice site, insertion, translation start site, and nonstop mutation. The missense mutation was the most obvious. We also found that single nucleotide polymorphisms (SNP) were more frequent than insertions or deletion and the most common single nucleotide variant (SNV) was C > T (Li et al., 2011). The number of altered bases in each sample was counted and the mutation types were plotted in a box plot. The 10 most prevalent mutated genes in LCC and RCC were shown with ranked percentages (Figs. S4 and S5). The Mann–Whitney test was used to compare the TMB of LCC and RCC, and the results showed that the RCC had a higher TMB (Fig. 9A). The mutation information of each sample in LCC and RCC was graphed in a waterfall plot (Figs. 9B–9C), which showed that the mutation frequency of RCC was generally higher than that of LCC. *APC*, *TP53*, *TTN*, and *KRAS* mutations were present in both LCC and RCC (Cappell, 2008). We found that *BRAF* mutations were more pronounced in RCC, and *APC* mutations were significantly higher in LCC. The higher immune infiltration and the higher *BRAF* mutation in RCC suggested that RCC is closely related to MSI. The study of Lochhead P et al. showed that BRAF mutations in colorectal cancer were linked to MSI through the methylation of CIMP and MLH1 promoter methylation (Lochhead et al., 2013). This is consistent with results from previous research (Popescu et al., 2021). The high *APC* mutation in LCC suggests that it may be related to the inactivation of the Wnt pathway (Faux et al., 2021). LINC02418 has been shown to be a tumor driver in colon cancer (Tian et al., 2020), and whether there is an inherent relationship between LINC02418 and the mutated gene has not been investigated. The association between the mutated gene and LINC02418 may become the direction of future research.

The correlation analysis of top 25 mutated genes in LCC and RCC was conducted using the maftools package. Molecular interactions were more frequent in RCC than in LCC (Figs. 9D –9E ). In RCC, the co-occurrence of *APC* and *KRAS* and the mutually exclusive relationship of *BRAF* with *APC* and *KRAS* further indicated a potential relationship with CIN and CIMP (Issa, 2008).The different molecular mechanisms of RCC and LCC suggest that they may require different therapeutic approaches and prognoses.

## Discussion

Colon cancer is one of the most common malignant tumors of the digestive system. Colon cancer can be defined as a left-sided or right-sided cancer according to the primary location of the tumor. The primary site of left-side colon cancer includes the splenic flexure, descending colon, and sigmoid colon. The right-side colon cancer includes the cecum, ascending colon, and hepatic flexure. The literature shows that prognosis of the left-side colon cancer is better than that of the right-side (Klose et al., 2020), and the survival rate is higher. Therefore, we need a better understanding of the classification of clinical subtypes of colon cancer.

We used the ssGSEA algorithm to obtain scores for 29 immune cell types and immune-related functions. We observed a high level of immune function in our analysis of RCC. This is manifested in *PD-L1* and *HLA* class I genes as well as high immune cell infiltration. Previous studies have shown that the changes of *HLA* class I genes and the high expression of *PD-L1* are both closely related to RCC (Kanno et al., 2020). We also observed that the immune score was significantly different between the two groups; the RCC presented with a higher immune score. This is consistent with previous reports (Pentheroudakis et al., 2015). The same is true for *HLA* family genes and immune checkpoint related gene expression and the abundance of immune cell infiltrations. Our results suggest that the RCC has more immune infiltration than the LCC, while RCC has a worse prognosis than LCC. TMB analysis showed that RCC with high immunity had a higher mutational burden than LCC, suggesting the existence of immune-evasive mutations and immune escape. Our comprehensive analysis found that the level of immune cell infiltration should not be the only determinant of prognosis.

The genetic factors of colorectal cancer include chromosome instability (CIN) and microsatellite instability (MSI) (Kaiser, Meckbach & Jacob, 2014). DNA mismatch repair (MMR) gene mutations or modifications may lead to a lack of MMR proteins, referred to as “microsatellite instability” (MSI), which can detect the rise or decline in the number of repeat sequences in tumor tissues and is caused by a repetitive sequence of insertions or deletions in the DNA. We found that *APC* mutations were significantly present in LCC, and *BRAF* mutations were significantly present in RCC. *APC* is a multifunctional gene, whose mutation is often associated with chromosome instability (CIN) (Hoevenaar et al., 2020), and plays an important role in the regulation of the Wnt signaling pathway. *APC* regulates the Wnt pathway by controlling the formation of β-catenin/Tcf, a nuclear complex that initiates Wnt target gene transcription (Raji, Sasikumar & Jacob, 2018). Both CIMP and *BRAF* mutations are closely related to RCC. Moreover, CIMP is often associated with an increased risk of malignant transformation, and *BRAF* mutations are suggestive of MSI. A number of studies have suggested that the occurrence of RCC is closely related to MSI. However, that RCC tends to have a poor prognosis despite its high MSI is contrary to previous studies (Laghi et al., 2020) where a high MSI indicates a good prognosis. This may suggest that *BRAF* negatively impacts the occurrence of RCC. The high correlation between RCC and *BRAF* suggests that there may be other prognostic pathways in the occurrence of RCC that are worth exploring. Based on these results, it is reasonable to assume that the prognostic efficacy of MMR is weak. It is reasonable to believe that the combination of immunotherapy and the analysis of the related signaling pathways will have important significance in the future cancer therapy.

From the above results and the clinicopathological analysis of the right hemicolon (Kalantzis et al., 2020), LCC and RCC may be tumors of different properties and have different carcinogenic mechanisms. We constructed a 4-mRNA LCC prognostic signature and a 6-mRNA RCC prognostic signature. Among the key genes, *C1ORF105* was associated with a larger inter-adventitial common carotid artery diameter (ICCAD) (Harrison et al., 2013), and *FAM132b* can be increased by induction of erythrogenesis (Gurieva et al., 2017), suggesting that they may play an important role in the occurrence and development of cancer. The overexpression of *TNNT1* may play a role in the development of diffuse midline gliomas (DMGs) (Vitanza et al., 2020). *RspO4* can activate the Wnt/β-catenin signaling pathway and promote the progression of esophageal squamous cell carcinoma (Chai et al., 2020). Genetic variation in *KLRG2* may influence the aggressiveness of prostate cancer (Liu et al., 2011). *MiR-1254* targets *PAX5* to reduce *HIPPO* signal, thereby promoting the proliferation, migration, and invasion of HCC cells (Lu et al., 2021). *SLC22A31* was differentially expressed between LCC and RCC in a study based on sequencing data (Liang et al., 2018). Currently, there is no reported association between *OFCC1*, *Syngr3*, *CCDC160* and cancer. Additional studies on the mechanism of action of these key genes in LCC and RCC are needed. The model has been verified by an internal testing set, a complete set, and an external testing and has been validated as an independent prognostic indicator. Prognostic signatures were established for left-sided and right-sided colon cancers, and have been validated internally and externally. These signatures provide the basis for individualized treatment of left and right-sided colon cancers.

RCC has a more pronounced mutation landscape than LCC according to previous studies (Jensen, Villanueva & Loaiza-Bonilla, 2018). We found that the expression of mutated genes in LCC was more positively correlated, and the results were more significant than that of RCC. These results suggest that the classification of clinical subtypes of colon cancer may be of great significance for the determination of clinical diagnosis and treatment in the future.

## Conclusions

We observed significant differences in the clinical characteristics, immune microenvironment, transcriptomic differences, and single gene mutation differences in the multi-omics analysis of LCC and RCC, suggesting that the difference in gene expression can be analyzed and divided into different clinical subtypes to help the early clinical diagnosis and prognosis of colon cancer. Our results may provide individualized treatment options and better prognostic evaluation for patients with left-side or right-side colon cancer. The 4-mRNA LCC prognostic signature and 6-mRNA RCC prognostic signature may provide a basis for personalized treatment of colon cancer. Further clinical testing is required to validate our results.

## Supplemental Information

10.7717/peerj.11433/supp-1 Supplemental Information 1 Clinical features for the LCC and RCC patients in GSE39582 of GEO database. **Abbreviations:** LCC: left-side colon cancer; RCC: right-side colon cancer; GEO: the Gene Expression Omnibus.

10.7717/peerj.11433/supp-2 Supplemental Information 2 Differential expressed gene in LCC and RCC.

10.7717/peerj.11433/supp-3 Supplemental Information 3 GO enrichment results of differentially expressed genes in LCC and RCC.

10.7717/peerj.11433/supp-4 Supplemental Information 4 KEGG enrichment results of differentially expressed genes in LCC and RCC.

10.7717/peerj.11433/supp-5 Supplemental Information 5 The univariate and multivariate Cox analysis for risk signature and clinical characteristics in LCC. **Abbreviations:** CI, confidence interval; HR, hazard ratio.

10.7717/peerj.11433/supp-6 Supplemental Information 6 The univariate and multivariate Cox analysis for risk signature and clinical characteristics in RCC. **Abbreviations:** CI, confidence interval; HR, hazard ratio.

10.7717/peerj.11433/supp-7 Supplemental Information 7 Comparison of survival rates of LCC and RCC in different clinical characteristics. The K-M survival curve of different clinical characteristics including (a)Age < 65,(b)Stage I&Stage II,(c)T1&T2,(e)N0,(E)Female

10.7717/peerj.11433/supp-8 Supplemental Information 8 Visualization of the 4-mRNA LCC prediction signature in the testing set and the total set. (a-b) Patients of high risk group(red dots) and low risk group(green dots), and the distribution of their corresponding riskscore.(c-d)Patients in high-risk group(red dots) and low-risk group (green dots), and their corresponding survival status.(e-f)Discrimination of the expression of 4-mRNA in signature between high-risk group and low-risk group, as revealed by a heatmap.

10.7717/peerj.11433/supp-9 Supplemental Information 9 Visualization of the 6-mRNA RCC prediction signature in the testing set and the total set. (a-b) Patients of high-risk group(red dots) and low-risk group(green dots), and the distribution of their corresponding riskscore. (c-d) Patients in high-risk group(red dots) and low-risk group (green dots), and their corresponding survival status. (e-f) Discrimination of the expression of 6-mRNA in signature between high-risk group and low-risk group, as revealed by a heatmap.

10.7717/peerj.11433/supp-10 Supplemental Information 10 The landscape of frequently mutated genes LCC. (a) Classifcation and frequency of mutation types.(b) Frequency of variant types (c) Frequency of SNV classes. (d-e) Tumor mutation burden in specific samples (f) The top 10 mutated genes

10.7717/peerj.11433/supp-11 Supplemental Information 11 The landscape of frequently mutated genes RCC. (a) Classifcation and frequency of mutation types. (b) Frequency of variant types (c) Frequency of SNV classes. (d-e) Tumor mutation burden in specific samples (f) The top 10 mutated genes

## Abbreviations

LCC: left-side colon cancer
RCC: right-side colon cancer
TCGA: The Cancer Genome Atlas
ssGSEA: single sample gene set enrichment analysis
HLA: human leukocyte antigen
DAVID: the Database for Annotation, Visualization and Integrated Discovery
GO: Gene Ontology
KEGG: Kyoto Encyclopedia of Genes and Genomes
K–M: Kaplan–Meier
ROC: receiver operating characteristic
GEO: the Gene Expression Omnibus
OS: Overall survival
SNP: single nucleotide poly-morphism
SNV: single nucleotide variants

## Additional Information and Declarations

### Data Availability

The following information was supplied regarding data availability: The raw data is available at TCGA-COAD and GSE39582.
