# Supplementary material for: Analysis of multi-omics differences in left-side and right-side colon cancer
Source: PeerJ. 2021 May 12;9:e11433. doi: 10.7717/peerj.11433 (PMC8123232; doi:10.7717/peerj.11433)
Supplement: Supplemental Information 1 — Abbreviations: LCC: left-side colon cancer; RCC: right-side colon cancer; GEO: the Gene Expression Omnibus. [file peerj-09-11433-s001.docx]

**Table S1 Clinical features for the LCC and RCC patients in GSE39582 of GEO database**

| **Parameters** | **LCC patients**  **(n = 342)** | **RCC patients**  **(n = 224)** |
| --- | --- | --- |
| **Age,y** |  |  |
| ≤65 | 158 | 64 |
| >65 | 184 | 160 |
| **Gender** |  |  |
| Male | 194 | 116 |
| Female | 148 | 108 |
| **pT** |  |  |
| T1-2 | 43 | 13 |
| T3-4 | 283 | 203 |
| unknow | 16 | 8 |
| **pN** |  |  |
| N0 | 188 | 114 |
| N1-2 | 134 | 98 |
| unknow | 20 | 12 |
| **pM** |  |  |
| M0 | 285 | 197 |
| M1 | 42 | 19 |
| unknow | 15 | 8 |
| **pStage** |  |  |
| Stage I-II | 183 | 114 |
| Stage III-IV | 157 | 108 |
| unknow | 2 | 2 |
| **Survival** |  |  |
| Alive | 225 | 146 |
| Dead | 117 | 74 |
| unknow | 0 | 4 |

**Abbreviations:**LCC:left-side colon cancer;RCC:right-side colon cancer;GEO:the Gene Expression Omnibus.
