# Supplementary material for: Analysis of multi-omics differences in left-side and right-side colon cancer
Source: PeerJ. 2021 May 12;9:e11433. doi: 10.7717/peerj.11433 (PMC8123232; doi:10.7717/peerj.11433)
Supplement: Supplemental Information 5 — Abbreviations: CI, confidence interval; HR, hazard ratio. [file peerj-09-11433-s005.docx]

**Table S5 The univariate and multivariate Cox analysis for risk signature and clinical characteristics in LCC**

| Factors | Univariate Cox analysis | | | | Multivariate Cox analysis | | | |
| --- | --- | --- | --- | --- | --- | --- | --- | --- |
|  |  | 95%CI | |  |  | 95%CI | |  |
|  | HR | Low | High | P Value | HR | Low | High | P Value |
| gender | 1.071 | 0.492 | 2.330 | 0.863 | 0.651 | 0.234 | 1.809 | 0.410 |
| pT | 1.936 | 0.812 | 4.612 | 0.136 | 1.428 | 0.375 | 5.434 | 0.601 |
| pN | 2.396 | 1.440 | 3.986 | 0.001 | 0.703 | 0.223 | 2.211 | 0.546 |
| pM | 3.477 | 1.478 | 8.182 | 0.004 | 0.459 | 0.038 | 5.576 | 0.541 |
| pStage | 2.298 | 1.432 | 3.689 | 0.001 | 4.698 | 0.673 | 32.786 | 0.119 |
| Age | 1.023 | 0.990 | 1.057 | 0.180 | 1.036 | 0.991 | 1.083 | 0.117 |
| riskScore | 1.126 | 1.079 | 1.174 | <0.001 | 1.134 | 1.079 | 1.191 | <0.001 |

**Abbreviations:** CI, confidence interval; HR, hazard ratio.
