# Supplementary material for: Analysis of multi-omics differences in left-side and right-side colon cancer
Source: PeerJ. 2021 May 12;9:e11433. doi: 10.7717/peerj.11433 (PMC8123232; doi:10.7717/peerj.11433)
Supplement: Supplemental Information 6 — Abbreviations: CI, confidence interval; HR, hazard ratio. [file peerj-09-11433-s006.docx]

**Table S5 The univariate and multivariate Cox analysis for risk signature and clinical characteristics in RCC**

| Factors | Univariate Cox analysis | | | | Multivariate Cox analysis | | | |
| --- | --- | --- | --- | --- | --- | --- | --- | --- |
|  |  | 95%CI | |  |  | 95%CI | |  |
|  | HR | Low | High | P Value | HR | Low | High | P Value |
| gender | 1.142 | 0.664 | 1.967 | 0.631 | 0.937 | 0.505 | 1.739 | 0.837 |
| pT | 3.366 | 2.007 | 5.647 | <0.001 | 2.228 | 1.020 | 4.869 | 0.045 |
| pN | 1.873 | 1.394 | 2.517 | <0.001 | 1.085 | 0.576 | 2.044 | 0.799 |
| pM | 4.895 | 2.594 | 9.239 | <0.001 | 0.893 | 0.213 | 3.736 | 0.876 |
| pStage | 2.294 | 1.659 | 3.173 | <0.001 | 1.906 | 0.650 | 5.588 | 0.240 |
| Age | 1.034 | 1.007 | 1.062 | 0.013 | 1.043 | 1.013 | 1.074 | 0.005 |
| riskScore | 1.002 | 1.001 | 1.003 | <0.001 | 1.002 | 1.000 | 1.003 | 0.009 |

**Abbreviations:** CI, confidence interval; HR, hazard ratio.
