# Supplementary figures and images for: Analysis of multi-omics differences in left-side and right-side colon cancer
Source: PeerJ. 2021 May 12;9:e11433. doi: 10.7717/peerj.11433 (PMC8123232; doi:10.7717/peerj.11433)

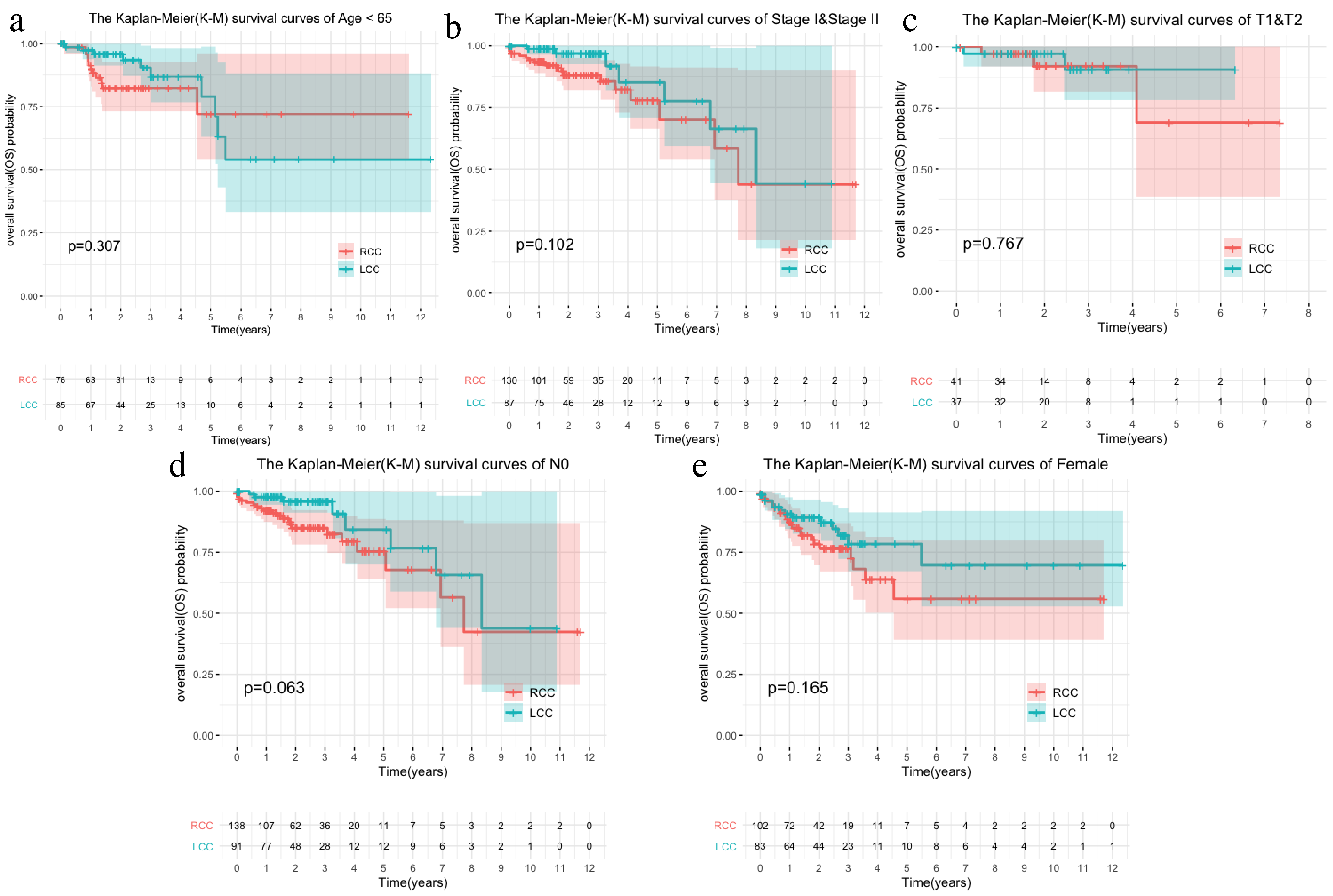

Supplement: Supplemental Information 7 — The K-M survival curve of different clinical characteristics including (a)Age < 65,(b)Stage I&Stage II,(c)T1&T2,(e)N0,(E)Female [file peerj-09-11433-s007.png]

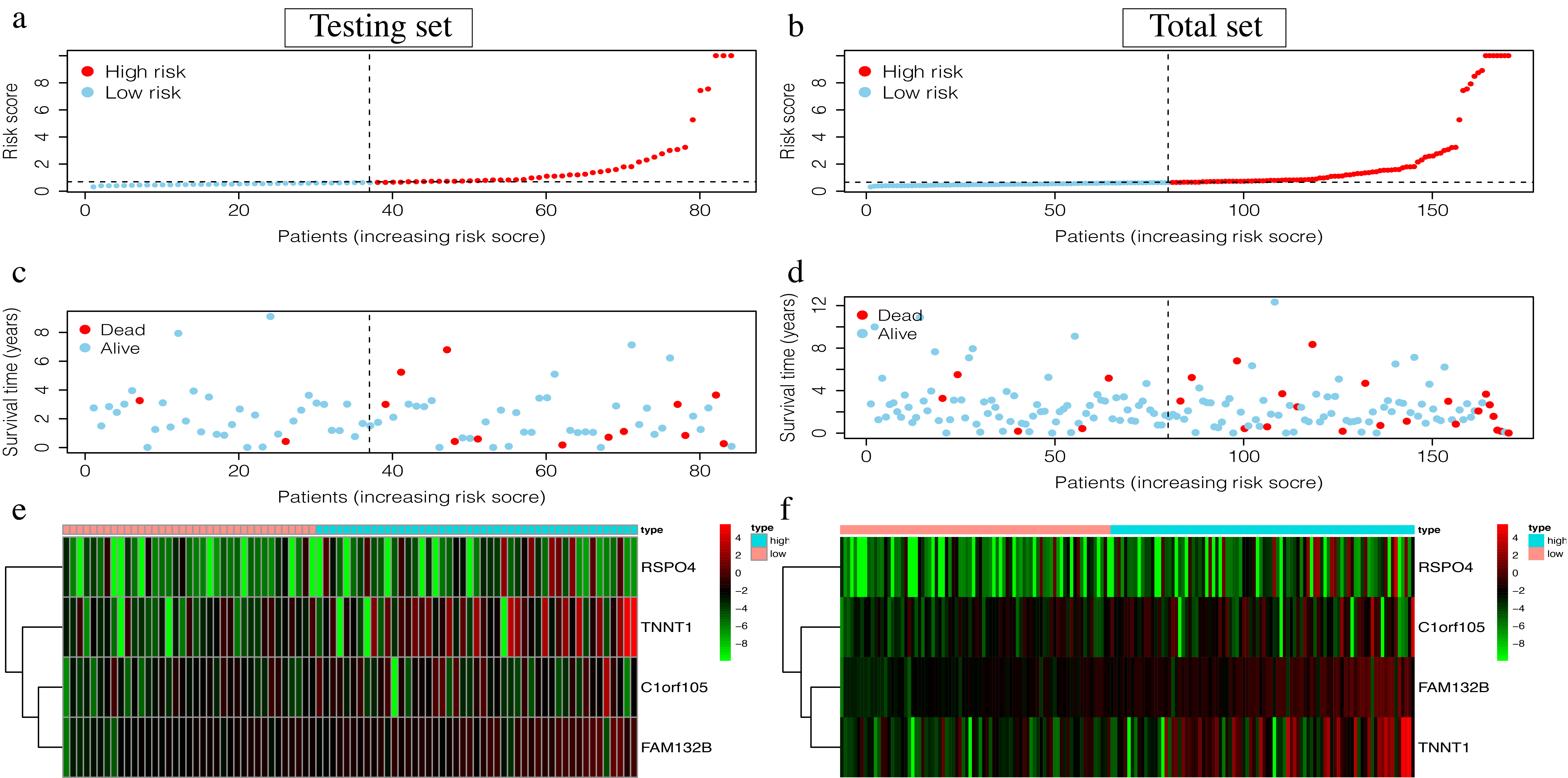

Supplement: Supplemental Information 8 — (a-b) Patients of high risk group(red dots) and low risk group(green dots), and the distribution of their corresponding riskscore.(c-d)Patients in high-risk group(red dots) and low-risk group (green dots), and their corresponding survival status.(e-f)Discrimination of the expression of 4-mRNA in signature between high-risk group and low-risk group, as revealed by a heatmap. [file peerj-09-11433-s008.png]

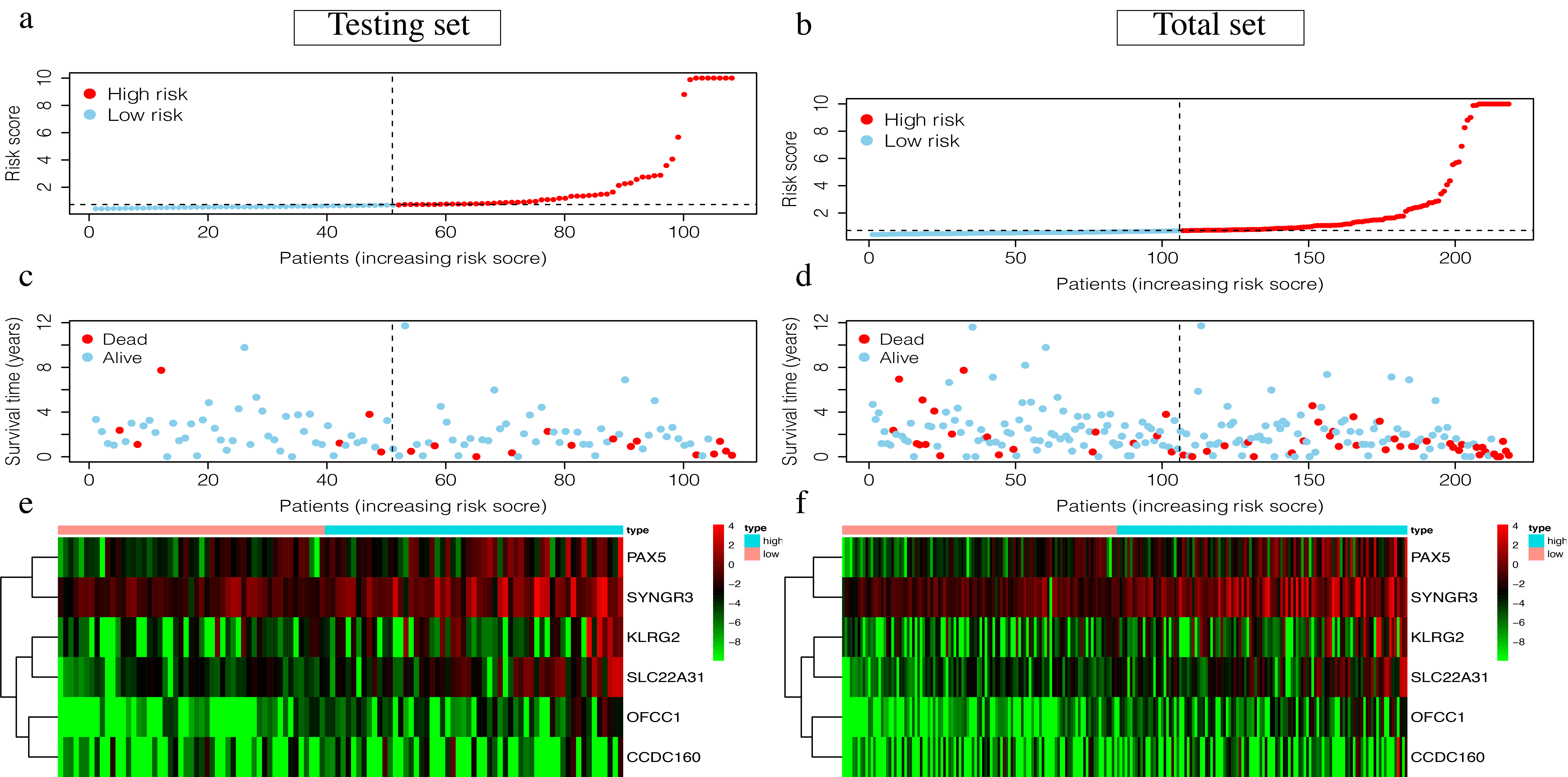

Supplement: Supplemental Information 9 — (a-b) Patients of high-risk group(red dots) and low-risk group(green dots), and the distribution of their corresponding riskscore. (c-d) Patients in high-risk group(red dots) and low-risk group (green dots), and their corresponding survival status. (e-f) Discrimination of the expression of 6-mRNA in signature between high-risk group and low-risk group, as revealed by a heatmap. [file peerj-09-11433-s009.png]

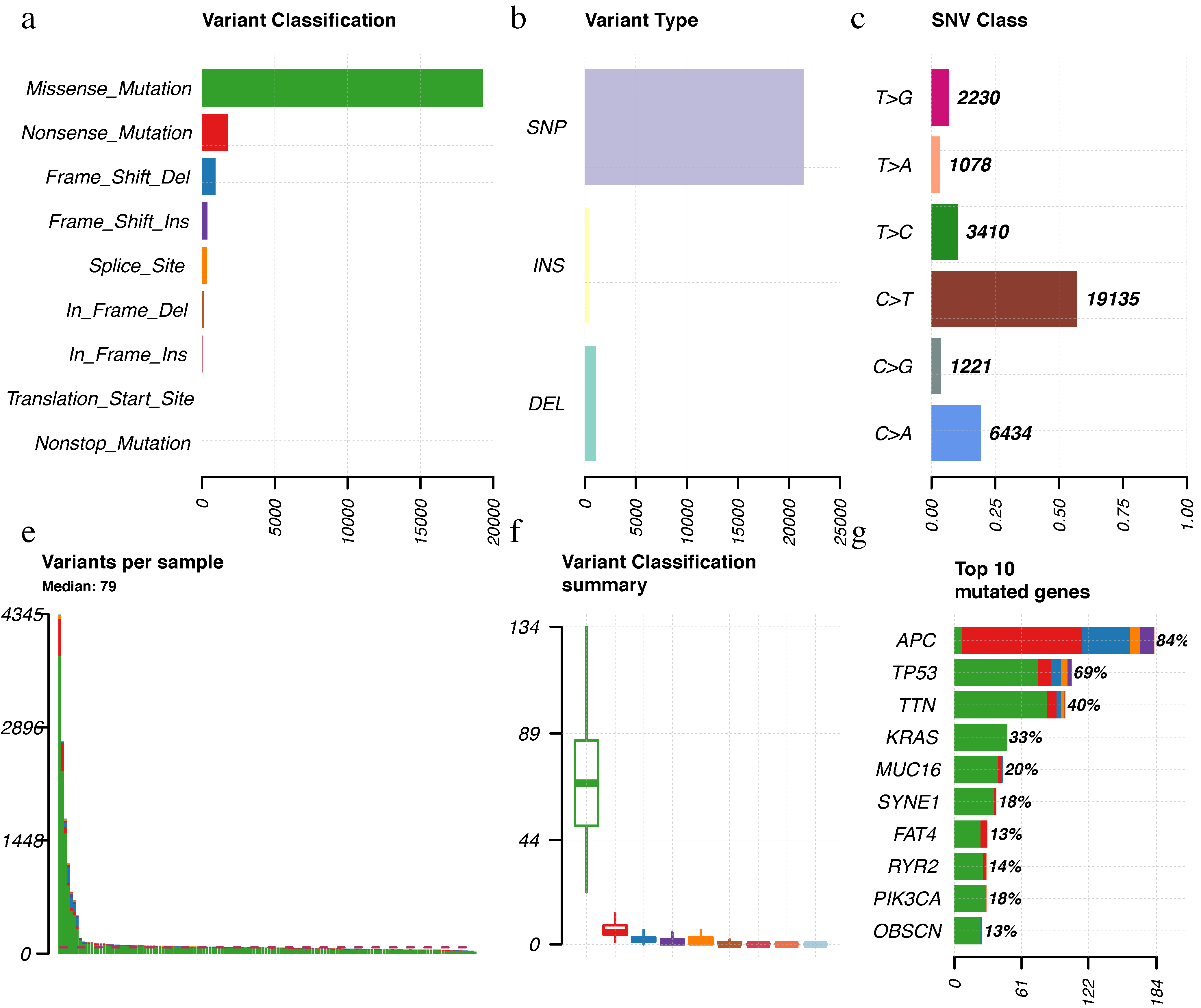

Supplement: Supplemental Information 10 — (a) Classifcation and frequency of mutation types.(b) Frequency of variant types (c) Frequency of SNV classes. (d-e) Tumor mutation burden in specific samples (f) The top 10 mutated genes [file peerj-09-11433-s010.png]

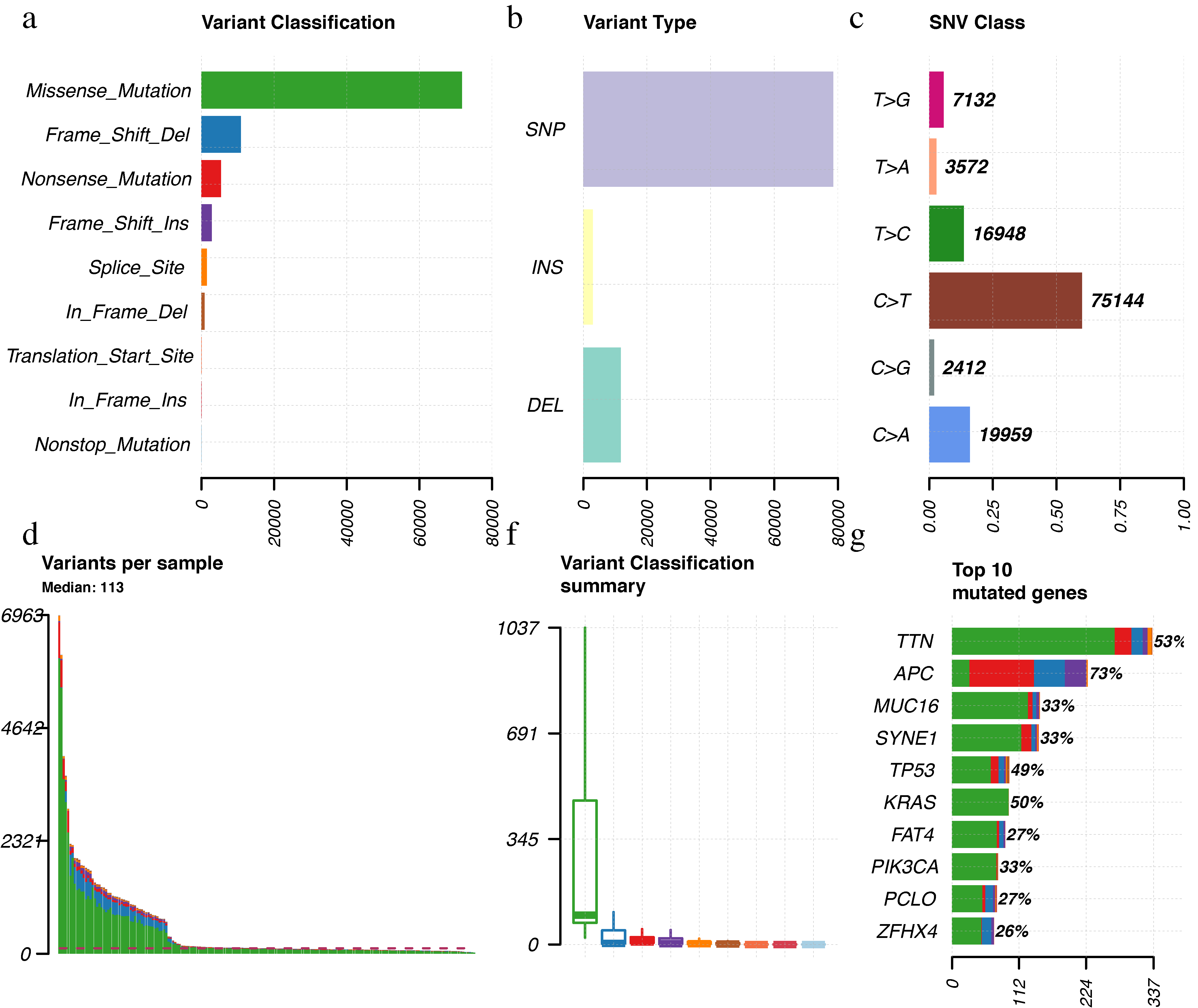

Supplement: Supplemental Information 11 — (a) Classifcation and frequency of mutation types. (b) Frequency of variant types (c) Frequency of SNV classes. (d-e) Tumor mutation burden in specific samples (f) The top 10 mutated genes [file peerj-09-11433-s011.png]
